# Development of a Tool to Measure Student Perceptions of Equity and Inclusion in Medical Schools

**DOI:** 10.1001/jamanetworkopen.2024.0001

**Published:** 2024-02-21

**Authors:** Dowin Boatright, Mytien Nguyen, Katherine Hill, David Berg, Laura Castillo-Page, Nientara Anderson, Victoria Agbelese, Shruthi Venkataraman, Somnath Saha, Stephen C. Schoenbaum, Regina Richards, Ayana Jordan, Emmanuella Asabor, Marney A. White

**Affiliations:** 1 Department of Emergency Medicine, New York University Grossman School of Medicine, New York; 2 Yale School of Medicine, New Haven, Connecticut.; 3 Department of Psychiatry, Yale School of Medicine, New Haven, Connecticut; 4 The National Academies of Sciences, Engineering, and Medicine; 5 Department of Immunobiology, Yale School of Medicine, New Haven, Connecticut; 6 Section of General Internal Medicine, Johns Hopkins University School of Medicine, Baltimore, Maryland; 7 Josiah Macy Jr Foundation, New York, New York; 8 Office of Diversity, Equity, Inclusion and Community Engagement, University of Colorado Anschutz Medical Campus, Aurora; 9 Department of Psychiatry, New York University Grossman School of Medicine; 10 Department of Social and Behavioral Sciences, Yale School of Public Health, New Haven, Connecticut

## Abstract

**Importance:**

Creating an inclusive and equitable learning environment is a national priority. Nevertheless, data reflecting medical students’ perception of the climate of equity and inclusion are limited.

**Objective:**

To develop and validate an instrument to measure students’ perceptions of the climate of equity and inclusion in medical school using data collected annually by the Association of American Medical Colleges (AAMC).

**Design, Setting, and Participants:**

The Promoting Diversity, Group Inclusion, and Equity tool was developed in 3 stages. A Delphi panel of 9 members identified survey items from preexisting AAMC data sources. Exploratory and confirmatory factor analysis was performed on student responses to AAMC surveys to construct the tool, which underwent rigorous psychometric validation. Participants were undergraduate medical students at Liaison Committee on Medical Education–accredited medical schools in the US who completed the 2015 to 2019 AAMC Year 2 Questionnaire (Y2Q), the administrations of 2016 to 2020 AAMC Graduation Questionnaire (GQ), or both. Data were analyzed from August 2020 to November 2023.

**Exposures:**

Student race and ethnicity, sex, sexual orientation, and socioeconomic status.

**Main Outcomes and Measures:**

Development and psychometric validation of the tool, including construct validity, internal consistency, and criterion validity.

**Results:**

Delphi panel members identified 146 survey items from the Y2Q and GQ reflecting students’ perception of the climate of equity and inclusion, and responses to these survey items were obtained from 54 906 students for the Y2Q cohort (median [IQR] age, 24 [23-26] years; 29 208 [52.75%] were female, 11 389 [20.57%] were Asian, 4089 [7.39%] were multiracial, and 33 373 [60.28%] were White) and 61 998 for the GQ cohort (median [IQR] age, 27 [26-28] years; 30 793 [49.67%] were female, 13 049 [21.05%] were Asian, 4136 [6.67%] were multiracial, and 38 215 [61.64%] were White). Exploratory and confirmatory factor analyses of student responses identified 8 factors for the Y2Q model (faculty role modeling; student empowerment; student fellowship; cultural humility; faculty support for students; fostering a collaborative and safe environment; discrimination: race, ethnicity, and gender; and discrimination: sexual orientation) and 5 factors for the GQ model (faculty role modeling; student empowerment; faculty support for students; discrimination: race, ethnicity, and gender; and discrimination: sexual orientation). Confirmatory factor analysis indicated acceptable model fit (root mean square error of approximation of 0.05 [Y2Q] and 0.06 [GQ] and comparative fit indices of 0.95 [Y2Q] and 0.94 [GQ]). Cronbach α for individual factors demonstrated internal consistency ranging from 0.69 to 0.92 (Y2Q) and 0.76 to 0.95 (GQ).

**Conclusions and Relevance:**

This study found that the new tool is a reliable and psychometrically valid measure of medical students’ perceptions of equity and inclusion in the learning environment.

## Key Points

**Question:**

Is it possible to develop a tool to assess students’ perceptions of the climate of equity and inclusion in US doctor of medicine–granting institutions?

**Findings:**

In this cohort study of 116 904 medical undergraduate students, a new tool demonstrated acceptable internal consistency, construct validity, and criterion validity as a measure of equity and inclusion.

**Meaning:**

These findings suggest that this tool is a reliable, psychometrically valid, and useful measure of medical students’ perceptions of equity and inclusion in the learning environment.

## Teaser

This cohort study developed and validated an instrument to measure students’ perceptions of the climate of equity and inclusion in medical schools.

## Introduction

Creating an inclusive and equitable learning environment has been a longstanding priority for national medical organizations including the Association of American Medical Colleges (AAMC),^1^ the American Medical Association,^2^ the National Medical Association,^3^ and the National Academies of Sciences.^4^ Yet, despite a national sense of urgency, data reflecting medical students’ perception of the climate of equity and inclusion are limited.

Our limited understanding of the climate of equity and inclusion creates several problems that have direct implications for the health care workforce. First, without clear, concise, and recurrent data, we cannot precisely quantify disparities in equity and inclusion that exist among medical students from different backgrounds. Second, we are neither able to gauge the immediate impact of these disparities on student well-being and achievement nor downstream impacts on the diversity of the physician workforce. Third, we lack the ability to develop evidence-based interventions to address disparities related to equity and inclusion in the learning environment.

These limitations are important because physicians from diverse backgrounds make important contributions to patient care,^5,6,7,8,9^ and a deleterious learning environment could limit physician workforce diversity.^10,11,12,13,14^ Consequently, we developed a new tool to measure students’ perceptions of the climate of equity and inclusion in medical school using data collected annually by the AAMC. In this article, we report on the development and psychometric validation of the tool as a sustainable, scalable, and generalizable measure of equity and inclusion in doctor of medicine (MD)–granting institutions.

## Methods

The development and validation of the tool, Promoting Diversity, Group Inclusion and Equity (PRODIGIE), proceeded through several stages. First, a Delphi panel was convened to identify survey items reflecting students’ perceptions of the climate of equity and inclusion in the medical learning environment from preexisting AAMC data sources. Next, 5 years of student responses to these items were obtained from the AAMC and used for analysis. Exploratory factor analysis (EFA) was performed on item responses to construct the tool. The tool then underwent psychometric validation. The study followed the Strengthening the Reporting of Observational Studies in Epidemiology (STROBE) reporting guideline. This study was approved by the Yale University institutional review board. Informed consent was not required because the analysis was conducted on a deidentified dataset.

### Tool Development

#### Delphi Panel Members

The Delphi panel included a team of 9 experts in diversity, equity, inclusion, medical education, and psychometrics that was diverse in terms of race, ethnicity, sex, career stage, and professional background (D.B., K.H., D.B., L.C.P., S.S., S.C.S., R.R., A.J., and E.A.). Members included physicians, PhDs in education and organizational psychology, and medical students.

#### Defining Equity and Inclusion

Before Delphi rounds began, Delphi panel members met to reflect upon and determine definitions for equity and inclusion that would be used throughout the Delphi process. Equity was defined as ensuring that everyone has access to the same opportunities. Inclusion was defined as all individuals feeling respected, supported, and valued.

#### Tool Item Selection

Panel members were given copies of all the AAMC medical student survey instruments, which included the Year 2 Questionnaire (Y2Q) and the Graduation Questionnaire (GQ). They were also given copies of the American Medical College Application Service and Electronic Residency Application Service instruments. Each year, survey and application data are collected by the AAMC and represent a comprehensive reflection of students’ self-reported demographic data and lived experiences during medical school.

Item selection for the tool was performed over the course of 3 Delphi panel rounds. During each round, panelists reviewed documents and provided recommendations on survey items for inclusion before meeting as a group. Subsequently, panelists met to discuss the survey questions identified. After each round, a summary document of results from the prior round was created and distributed to panelists. This process had several benefits. Allowing experts to provide input before meeting afforded panel members anonymity when responding and reduced limitations commonly inherent to group interactions, including the influences of dominant personalities and the pressure to conform.^15^

Controlled quantitative and qualitative feedback between rounds allowed each expert to generate additional insights and the ability to clarify information from previous rounds. Meetings permitted the benefits of face-to-face interaction to exchange information and resolve uncertainties. During the third round of the Delphi panel, members focused on resolving any conflicts regarding survey items for inclusion in the tool. Survey items for which consensus was reached were included. As is common in the Delphi process, consensus was defined as when 75% of panelists were in agreement.^16^

#### Final Survey Item List

The Delphi panel identified 146 survey items. Survey items were chosen from the AAMC’s Y2Q (80 items) and GQ (66 items).

#### Study Population and Data Sources

Student responses were obtained for all survey items identified by the Delphi Panel. Student responses came from the 2015 to 2019 administrations of the AAMC Y2Q and the administrations of 2016 to 2020 AAMC GQ. Demographic data for student self-reported race, ethnicity, sex, sexual orientation, and socioeconomic data were abstracted from the AAMC’s data warehouse and the AAMC’s Applicant Matriculant Data File. Participants selected race and ethnicity from the following categories: Asian; American Indian, Alaska Native, Native Hawaiian, or Other Pacific Islander; Black or African American; Hispanic White; multiracial; and unknown/other. All students who reported Hispanic ethnicity were categorized as Hispanic regardless of race. Students were considered to come from low-income backgrounds if they indicated that they received Pell grants and/or state or federal financial assistance. Consistent with prior literature, marginalized identity was defined as a sociodemographic identity known to have been historically minoritized or discriminated against in medicine (female sex; non-White race; Hispanic ethnicity; lesbian, bisexual, or gay sexual orientation; and low-income status).^10,17,18^

The initial Y2Q sample included 65 437 students. Each medical school was deidentified by the AAMC. To avoid the passive reidentification of a medical school, the investigative team did not receive institutional data from survey respondents from historically black colleges or universities (HBCU), Puerto Rican medical schools, or medical schools founded after 2014. Overall, 10 531 students (16.1%) were excluded: 2376 (3.6%) were excluded because they attended HBCUs or Puerto Rican medical schools or medical schools founded after 2014; 265 (0.4%) were missing race or ethnicity; 20 (0.03%) were missing sex; 6477 (9.9%) were missing sexual orientation; and 2303 (3.5%) were missing socioeconomic data. Compared with students excluded, a greater percentage of students included reported White race and female sex.

The initial GQ sample included 80 350 students. Overall, 18 352 (22.8%) were excluded due to missing sociodemographic data. A total of 3460 (4.3%) were excluded because they attended an HBCU, Puerto Rican medical school, or school founded after 2014. Of students excluded for missing demographic information, 248 (0.3%) were missing race or ethnicity, 7 (0.01%) were missing sex, 6532 (8.1%) were missing sexual orientation, and 9832 (12.2%) were missing socioeconomic data. Compared with students excluded, a greater percentage of students included reported White race and female sex.

Because we were most interested in gaining insight into the climate of equity and inclusion in the medical learning environment,^19^ subsequent EFA and confirmatory factor analysis (CFA) were limited to students that we felt a priori would be most acutely attuned to these issues due to a well-documented history of discrimination in academic medicine (male and female students who self-reported Black race; Hispanic/LatinX ethnicity; or lesbian, gay, or bisexual sexual orientation).^10,11,12,13,14,18^ These groups were chosen because studies with intersectional analyses of medical trainees’ experiences have shown that, while other demographic groups are exposed to bias and discrimination, individuals who identify as underrepresented in medicine, lesbian, gay, bisexual, or who have multiple marginalized identities often report the highest rates of bias, mistreatment, and discrimination.^10,17,18,20^ To generate the data sets for EFA and CFA, we used the random selection function in SPSS version 26 (IBM). The sample analyzed from the Y2Q totaled 30 571 (EFA: 14 544; CFA: 16 207) and that from the GQ totaled 36 266 (EFA: 18 307; CFA: 17 959).

#### Exploratory Factor Analysis

Exploratory factor analyses^21,22^ were conducted on the GQ and Y2Q data sets separately to determine the extent to which the survey items represented underlying factors. We performed exploratory factor analysis for the year 2 and graduation time points separately because of the distinct preclinical and clinical environments that are often demarcated at the second year of medical education. Because we hypothesized that all or some of the latent factors were correlated, we used the common factors method of analysis using an oblique (PROMAX) rotation of the correlation matrices.^22^

Beginning with data from the GQ, data from approximately one-half of the sample were randomly selected and used to conduct the exploratory factor analysis. The Kaiser-Meyer-Olkin measure of sampling adequacy and the Bartlett test of sphericity were calculated to assess the appropriateness of the data for factor analysis. Items were retained if they had a factor loading of 0.4 or higher and if they loaded on only 1 factor. A parallel process was used to conduct an EFA on the Y2Q survey data.

#### Psychometric Validation

##### Confirmatory Factor Analysis

CFA was used to test the models identified through EFA procedures with the remaining approximate one-half of the samples from the GQ and Y2Q. We used the following conventions to test the adequacy of the models: root mean square error of approximation (RMSEA), comparative fit index (CFI), Tucker-Lewis Index (TLI), and standardized root mean squared residual (SRMR).^23,24,25^ Using standard conventions, RMSEA close to 0.06, a CFI and TFI close to 0.95, and SRMR less than 0.08 indicated good model fit.^26^

##### Internal Consistency

Internal consistency is an important component of reliability that indicates whether people give similar responses to items meant to measure the same factor.^27^ Internal consistency was evaluated via Cronbach α, calculated for each factor.

##### Criterion Validity

Criterion validity evaluates whether an instrument produces scores that correlate with outcomes in a manner consistent with theory.^28^ To examine the criterion validity of the tool, we conducted 2 tests.

First, we calculated mean tool scores for each medical school by only including responses from students who reported no marginalized identities (ie, students who reported being male sex, non-Hispanic White race, heterosexual, and not low-income). Then we calculated the scores for students who reported 1 marginalized identity, followed by 2 marginalized identities, 3 marginalized identities, and last, mean tool scores by medical school for students who reported 4 marginalized identities. We hypothesized that the mean medical school tool scores would decrease as students reported additional marginalized identities.

Second, we determined differences in mean tool scores between marginalized and nonmarginalized groups (female vs male; Asian, Black, Hispanic/LatinX vs White; LGB vs non-LGB; and low SES vs nonlow SES). We hypothesized that members of marginalized groups would have lower mean tool scores than their nonmarginalized peers.

Tool scores for individual factors identified through factor analysis were calculated as an average of all items comprising those factors. Because individual items may have originally contained scales of varying ranges, all individual items were standardized to a 5-point scale (range from 1-5). Overall medical school tool scores were calculated as a sum of all subscale scores to give equal weight across factors and normalized to range from 1-100 scale.

We compared the mean medical school tool scores calculated by the number of students’ marginalized identities using ANOVA with multiple comparison with Tukey adjustment. For all other identities, 2-tailed independent *t* tests were used to determine whether differences in mean scores between nonmarginalized and marginalized groups were statistically significant. Significance was set at a *P* value of less than .05.

Statistical analyses were performed using R version 4.1.2 and RStudio (R Project for Statistical Computing), SPSS version 28.0.0.0 (IBM), and STATA version 17 (StataCorp). Data were analyzed from August 2020 to November 2023.

## Results

The final GQ cohort included 61 998 students, of whom 30 793 (49.67%) were female, 4099 (6.61%) were LGB, 15 700 (25.32%) were from low-income backgrounds, 13 049 (21.05%) reported Asian race, 38 215 (61.6%) reported White race, and 4136 (6.67%) reported being multiracial. The final Y2Q cohort included 54 906 students, of which 29 208 (52.75%) were female, 4412 (7.97%) were LGB, 16 805 (30.35%) were from low-income backgrounds, 11 389 (20.57%) reported Asian race, 33 373 (60.28%) reported White race, and 4089 (7.39%) reported being multiracial (Table 1).

**Table 1.** Characteristics of Year 2 Questionnaire and Graduation Questionnaire Cohorts

Characteristic	Year 2 Questionnaire (n = 54 906)	Graduation Questionnaire (n = 61 998)
Sex		
Male	26 158 (47.25)	31 205 (50.33)
Female	29 208 (52.75)	30 793 (49.67)
Race and ethnicity		
American Indian, Alaska Native, Hawaiian Native, and Pacific Islander	133 (0.24)	127 (0.20)
Asian	11 389 (20.57)	13 049 (21.05)
Black	3078 (5.56)	2951 (4.76)
Hispanic	2301 (4.16)	2480 (4.00)
Multiracial	4089 (7.39)	4136 (6.67)
Other	1003 (1.81)	1040 (1.68)
White	33 373 (60.28)	38 215 (61.64)
Lesbian, gay, or bisexual		
No	50 954 (92.03)	57 899 (93.39)
Yes	4412 (7.97)	4099 (6.61)
Low income^a^		
No	38 561 (69.65)	46 298 (74.68)
Yes	16 805 (30.35)	15 700 (25.32)
No. of marginalized identities		
0	11 106 (20.23)	14 600 (23.55)
1	22 295 (40.61)	25 713 (41.47)
2	15 415 (28.08)	16 219 (26.16)
3	5732 (10.44)	5228 (8.43)
4	358 (0.65)	238 (0.38)

^a^ Low-income is defined as students who either received a Pell grant or state or federal financial assistance.

### Exploratory Factor Analysis

EFA of student responses from the GQ demonstrated that the Kaiser-Meyer-Olkin index (KMO) was 0.95 and the Bartlett test was significant, indicating that the data were appropriate for analysis. We identified 5 factors (faculty role modeling; student empowerment; faculty support for students; discrimination: race, ethnicity, and gender; and discrimination: sexual orientation) derived from GQ items accounting for 63.7% of the total variance (Table 2). The EFA of student responses from the Y2Q demonstrated that the KMO (0.94) and the Bartlett test (GQ: χ^2^_465_ = 323 418.4; *P* < .001; Y2Q: χ^2^_595_ = 217 428.3; *P* < .001) were appropriate for analysis. We identified 8 factors (faculty role modeling; student empowerment; student fellowship; cultural humility; faculty support of students; fostering a collaborative and safe environment; discrimination: race, ethnicity, and gender; and discrimination: sexual orientation) derived from Y2Q items accounting for 64.5% of the total variance (Table 2).

**Table 2.** Factor Loadings of the Year 2 Questionnaire and Graduation Questionnaire: Exploratory and Confirmatory Analyses^a^

Factor and Item	EFA	CFA	α
Year 2 Questionnaire			
Faculty role modeling			
Faculty respectful of house staff	0.84	0.84	0.89
Faculty respect patient confidentiality	0.83	0.70	
Faculty dress in a professional manner	0.81	0.64	
Faculty respectful of other health professions	0.68	0.78	
Faculty use professional language	0.65	0.64	
Faculty resolve conflicts respectfully	0.60	0.80	
Faculty respectful of diversity	0.49	0.77	
Student empowerment			
Students feel achievement	0.97	0.92	0.92
Students value themselves	0.85	0.90	
Students feel confident	0.78	0.85	
Student fellowship			
Students gather for informal activities	0.91	0.81	0.83
Students assist each other	0.77	0.79	
Students get to know each other well	0.71	0.76	
Cultural humility training			
Learn to work in disadvantaged communities	0.77	0.73	0.76
Learn tools to recognize their own bias	0.74	0.70	
Learn to communicate with people from different backgrounds	0.66	0.75	
Faculty support of students			
Teachers tell me I can perform well against high standards	0.52	0.49	0.69
Faculty helpful with nonacademic advice	0.43	0.73	
Faculty helpful with academic difficulty	0.41	0.76	
Fostering a collaborative and safe environment			
Faculty show empathy	0.77	0.87	0.82
Faculty provide constructive feedback	0.71	0.81	
Discrimination: race, ethnicity, and gender			
Denied opportunities based on race	0.61	0.56	0.71
Received lower grades based on race	0.61	0.69	
Received lower grades based on gender	0.61	0.67	
Denied opportunities based on gender	0.60	0.63	
Subjected to racially offensive remarks	0.59	0.47	
Subjected to sexist remarks	0.56	0.44	
Publicly belittled or humiliated	0.50	0.47	
Subjected to unwanted sexual advances	0.42	0.47	
Discrimination: sexual orientation			
Received lower grades based on sexual orientation	0.87	0.91	0.86
Asked to exchange sexual favors for rewards	0.70	0.90	
Denied opportunities based on sexual orientation	0.67	0.77	
Graduation Questionnaire			
Faculty role modeling			
Faculty respectful of patient dignity	0.89	0.84	0.95
Faculty respectful of other health professions	0.89	0.81	
Faculty respectful of house staff	0.85	0.8	
Faculty resolve conflicts respectfully	0.85	0.85	
Faculty listen to patients	0.84	0.84	
Faculty respectful of other specialties	0.79	0.76	
Faculty show empathy	0.78	0.86	
Faculty respectful of diversity	0.76	0.76	
Faculty respect patient confidentiality	0.72	0.67	
Faculty use professional language	0.7	0.65	
Faculty respectful of students	0.69	0.83	
Faculty provide constructive feedback	0.57	0.78	
Student empowerment			
Students feel achievement	0.96	0.94	0.94
Students value themselves	0.91	0.93	
Students feel confident	0.81	0.88	
Faculty support of students			
Faculty helpful with criticism	0.81	0.81	0.85
Faculty helpful with nonacademic advice	0.69	0.84	
Faculty helpful with academic difficulty	0.64	0.79	
Discrimination: race, ethnicity, and gender			
Denied opportunities based on race	0.42	0.43	0.76
Received lower grades based on race	0.83	0.76	
Received lower grades based on gender	0.79	0.73	
Denied opportunities based on gender	0.55	0.62	
Subjected to racially offensive remarks	0.53	0.55	
Subjected to sexist remarks	0.43	0.56	
Publicly belittled or humiliated	0.33	0.43	
Discrimination: sexual orientation			
Received lower grades based on sexual orientation	0.89	0.82	0.76
Asked to exchange sexual favors for rewards	0.86	0.83	
Subjected to offensive remarks based on sexual orientation	0.63	0.63	
Denied opportunities based on sexual orientation	0.53	0.50	
Been threatened with physical harm	0.45	0.47	

Abbreviations: CFA, confirmatory factor analysis; EFA, exploratory factor analysis.

^a^ EFA column displays the factor loadings of the exploratory factor analysis. CFA column denotes the factor loadings from the confirmatory analysis. A criterion of 0.45 was used as the cutoff for inclusion on a factor.

### Confirmatory Factor Analysis

Results indicated that the 5-factor model for the GQ was appropriate for the data. The RMSEA was 0.06, indicating a good fit. The CFI (0.94) and TLI (0.93) values also indicated acceptable fit, as did the SRMR (0.04). Additionally, results demonstrated the 8-factor model for the Y2Q was appropriate. The RMSEA was 0.05, indicating a very good fit. The CFI (0.95) and the TLI (0.94) also indicated acceptable fit, as did the SRMR of 0.04. All factor loadings were greater than 0.40 for both the 5-factor GQ model and the 8-factor Y2Q model, which is considered acceptable.^29^ Table 2 provides the individual items of the tool, with corresponding factor loadings from the EFA and CFA.

### Internal Consistency

For the GQ PRODIGIE model, Cronbach α for each factor ranged from 0.76 to 0.95 (Table 2). For the Y2Q model, Cronbach α for each factor ranged from 0.69 to 0.92 (Table 2). With the exception of 1 marginal Y2Q factor, Cronbach α for all factors was greater than 0.70, which is in the range considered acceptable.^30^

### Criterion Validity

Tool scores were calculated for 134 medical schools at the Y2Q time point and 129 schools for the GQ time point. At the GQ time point, the mean (SD) medical school tool score was 82.9 (2.5), with scores ranging from 77.1 to 91.3. For the Y2Q time point, the mean (SD) medical school tool score was 80.6 (2.5), and scores ranged from 74.6 to 88.3.

Mean (SD) medical school tool scores were greatest when only including students who reported no identities historically marginalized in medicine (82.3 [2.8] for Y2Q and 84.4 [2.7] for the GQ). As we restricted our analysis to medical students reporting additional identities historically marginalized in medicine, mean medical school tool scores decreased in a stepwise fashion as the number of marginalized identities reported by students increased. Mean (SD) medical school tool scores were 81.2 (2.6), 79.9 (2.8), 77.9 (3.9), and 76.5 (9.7) when restricting the analysis to students with 1, 2, 3, and 4 marginalized identities, respectively, for the Y2Q (Bartlett statistic, 185.1; *P* < .001) and 83.6 (2.6), 82.4 (2.7), 80.8 (3.5), and 79.1 (9.0) for the GQ (Bartlett statistic, 104.9; *P* < .001) (Figure).

**Figure.**
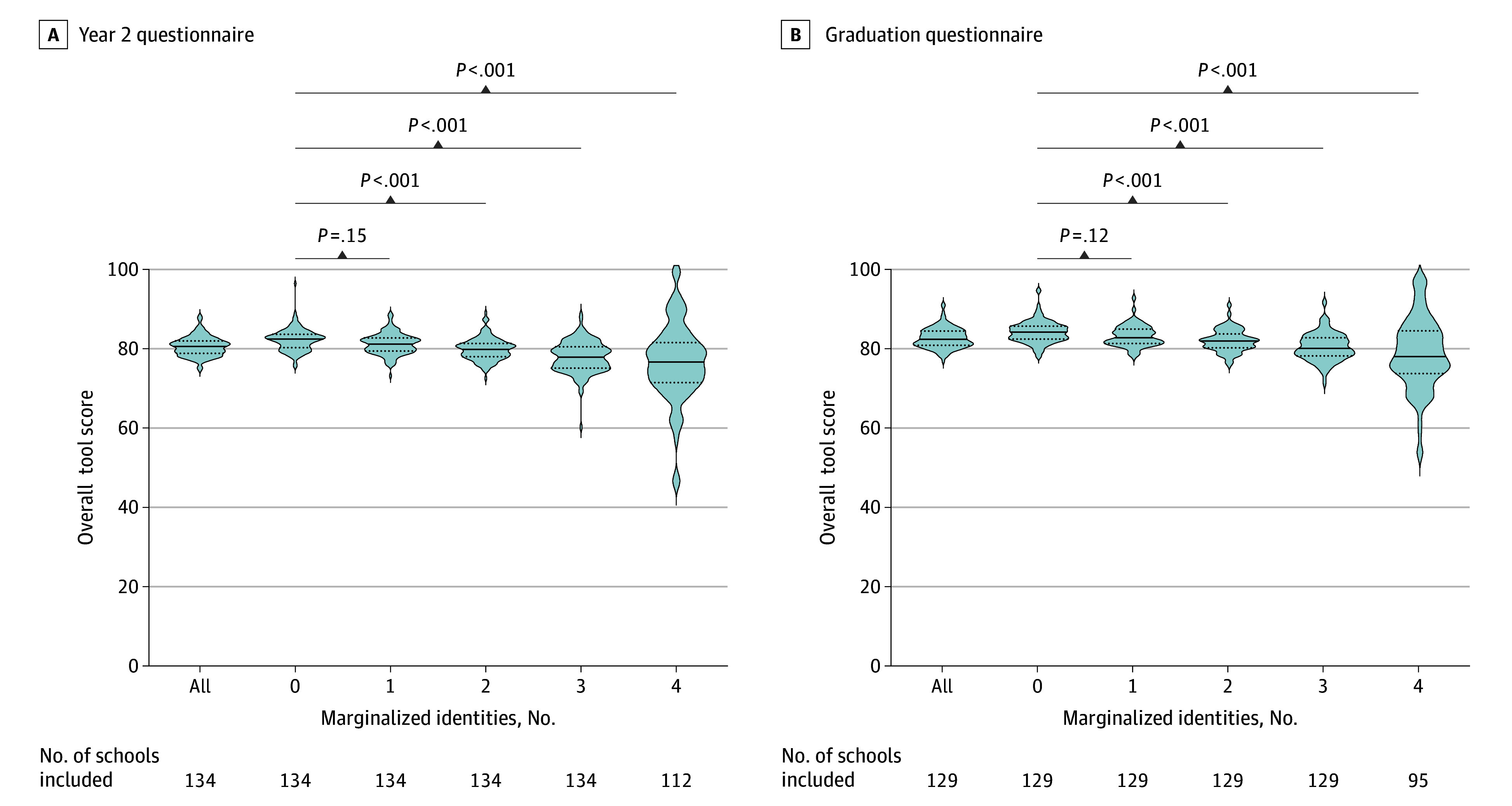
School-Averaged Overall Promoting Diversity, Group Inclusion, and Equity Tool Scores Across Groups of Multiple Marginalized Identities Average overall Year 2 Questionnaire (A) and Graduation Questionnaire (B) tool scores by institution for all students and across students who identified with 0, 1, 2, 3, or 4 marginalized identities. Overall, 22 of 134 (16.4%) and 34 of 129 (26.3%) medical schools did not have any student identifying with 4 concurrent marginalized identities in the Year 2 Questionnaire and Graduate Questionnaire, respectively.

Mean (SD) tool scores for female students were lower than scores for male students (80.05 [10.1] vs 81.21 [10.8]; η^2^ = 0.003; *P* < .001); scores for Asian (79.68 [10.21]), Black (77.62 [11.15]), and Hispanic (79.94 [10.99]) students reported lower scores than White students (81.28 [9.73]) (η^2^ = 0.008; *P* < .001); scores for LGB students were lower than scores for non-LGB students (78.17 [10.85] vs 80.81 [10.01]; η^2^ = 0.005; *P* < .001); and scores for students from low-income backgrounds were lower than scores from students not from low-income backgrounds (79.91 [10.69] vs 80.89 [9.83]; η^2^ = 0.001; *P* < .001) in analyzing scores from the Y2Q time point (Table 3). Similarly, mean (SD) tool scores calculated at the GQ time point were lower for female students compared with male students (81.82 [10.19] vs 83.32 [10.41]; η^2^ = 0.004; *P* < .001); lower for Asian (82.0 [10.86]), Black (80.56 [11.28]), and Hispanic (82.18 [10.96]) students compared with White students (82.97 [9.91]) (η^2^ = 0.003; *P* < .001); lower for LGB students compared with non-LGB students (79.64 [11.36] vs 82.77 [10.22]; η^2^ = 0.005; *P* < .001); and lower for students from low-income backgrounds compared with students not from low-income backgrounds (81.85 [11.05] vs 82.81 [10.06]; η^2^ = 0.001; *P* < .001, Table 3). Similar differences in scores were present in the mean tool scores among most individual factors for the Y2Q and GQ (eTable 1 and eTable 2 in Supplement 1).

**Table 3.** Year 2 and Graduation Questionnaire Average Overall Promoting Diversity, Group Inclusion, and Equity Factor Scores Across Students’ Sociodemographic Characteristics

Mean (SD)	Year 2 Questionnaire (n = 54 906)	η^2^	*P* value	Graduation Questionnaire (n = 61 998)	η^2^	*P* value
Total, mean (SD)	80.60 (10.11)	NA	NA	82.57 (10.33)	NA	NA
Sex						
Male	81.21 (10.08)	0.003	<.001	83.32 (10.41)	0.004	<.001
Female	80.05 (10.10)			81.82 (10.19)		
Race and ethnicity						
American Indian, Alaska Native, Hawaiian Native, and Pacific Islander	81.19 (11.50)	0.008	<.001	81.26 (12.94)	0.003	<.001
Asian	79.68 (10.21)			82.00 (10.86)		
Black	77.62 (11.15)			80.56 (11.28)		
Hispanic	79.94 (10.99)			82.18 (10.96)		
White	81.28 (9.73)			82.97 (9.91)		
Multiracial	80.44 (10.32)			82.55 (10.35)		
Other	79.20 (11.97)			81.40 (12.43)		
Lesbian, gay, or bisexual						
No	80.81 (10.01)	0.005	<.001	82.77 (10.22)	0.005	<.001
Yes	78.17 (10.85)			79.64 (11.36)		
Low income						
No	80.89 (9.83)	0.001	<.001	82.81 (10.06)	0.001	<.001
Yes	79.91 (10.69)			81.85 (11.05)		
No. of marginalized identities						
0	81.94 (9.44)	0.014	<.001	83.90 (9.72)	0.011	<.001
1	81.16 (9.83)			82.86 (10.08)		
2	79.89 (10.34)			81.78 (10.59)		
3	77.92 (10.96)			80.03 (11.41)		
4	76.45 (11.38)			77.54 (12.94)		

Abbreviation: NA, not applicable.

## Discussion

Our results demonstrate that the new tool is a reliable and psychometrically valid measure of medical students’ perceptions of equity and inclusion in the learning environment. The tool exhibited acceptable internal consistency overall and within individual factors. Consistent with our a priori assumptions and prior literature,^10,12,14,18^ students from historically marginalized backgrounds rated the medical learning environment less favorably than students from more privileged groups, supporting the tool’s criterion validity.

This new validated tool provides several benefits. It can provide a global assessment of the climate of equity and inclusion at all MD-granting institutions. Insight gained from the tool’s global assessment is complemented and bolstered by specific data present in individual tool factors, which reflect students’ perceptions of important facets of the learning environment, including faculty support of students, student fellowship, and discrimination.

The tool provides an assessment of equity and inclusion in the learning environment at 2 critical and distinct time points, which, for most schools, demarcate a transition from a classroom-based to a clinical learning environment. Because the tool can measure differences in the climate of equity and inclusion by student sociodemographic identity, it can quantify the experience of inequity and exclusion in undergraduate medical education, both nationally and at the level of individual medical schools.

The tool’s capabilities have important implications. The tool’s climate assessment can provide leaders and accreditors of MD-granting institutions recurrent, concise, and transparent data describing students’ experiences of equity and inclusion within their institutions. With these data, medical school leadership and medical school accreditors, such as the Liaison Committee on Medical Education, can, for the first time, have a comprehensive understanding of how the learning environment may differentially benefit some students more than others. Additionally, a global climate assessment can provide leaders of MD-granting institutions with national benchmarking data by which to compare their own institutional metrics.

Furthermore, medical school leadership can use institutional-level data to longitudinally gauge progress toward creating an equitable and inclusive learning environment using the tool for benchmarking. Individual tool factors can offer medical school leadership insight into specific aspects of the learning environment needing improvement and can inform evidence-based interventions to increase equity and inclusion.

Because the tool relies on existing surveys administered by the AAMC, it has none of the drawbacks of traditional climate surveys, such as participant survey fatigue. To facilitate and standardize tool score calculations, medical schools could partner with the AAMC to obtain school-level and national benchmarking data. The tool score reports could be aggregated over 3-year or 5-year time periods to ensure student anonymity, especially for students reporting multiple identities historically marginalized in medicine.

It is important to note that mean tool scores between marginalized and nonmarginalized groups are statistically significant, but differences and their associated effect sizes are small. Nevertheless, prior research has shown that small differences between groups, even with small effect sizes, can accumulate over time and have substantial influence on real world outcomes.^31,32,33,34,35^ Moreover, the size of differences between groups are similar to results found in other climate surveys used by the AAMC and in prior national studies.^36,37^ Future studies of the tool will examine how differences in tool scores influence disparities in consequential student outcomes in the learning environment, including attrition, successful placement into graduate medical education, burnout, and the receipt of academic awards.

### Limitations

Our study has limitations. Items selected for inclusion in the tool were limited to survey questions currently administered to medical students by the AAMC. Consequently, the tool may not capture all aspects of equity and inclusion in the learning environment. Nevertheless, as the AAMC expands its cadre of data collection instruments examining students’ experiences, the tool can be enhanced to capture these phenomena.

Creation of the tool relied on student responses to AAMC surveys. The AAMC’s GQ has a historical response rate of approximately 80%,^38^ and the AAMC’s Y2Q has a response rate of 59%.^39^ Prior studies have shown that students from marginalized backgrounds may be less likely than their peers to complete these surveys.^18^ Consequently, the tool may not capture the perspective of marginalized students in its entirety.

Historically, students from marginalized groups have higher rates of attrition in medical school than their counterparts.^40^ Tool results, especially at the GQ time point, may not fully reflect the degree of inequity and exclusion in the medical school learning environment secondary to student survival bias.

Additionally, the tool, in its current iteration, does not reflect the lived experience of medical students from all marginalized backgrounds. In particular, understanding how students reporting disabilities, religious beliefs, and nonbinary gender identities experience the learning environment will be critical to promote equity and inclusion in medicine.

## Conclusions

This study demonstrates that this new tool is a psychometrically valid and reliable measure of the climate of equity and inclusion in the medical school learning environment. Medical schools can use the tool to benchmark their students’ perception of equity and inclusion in the learning environment and identify areas for improvement to address disparities.

## Notes

Supplement 1. **eTable 1. **Year 2 Questionnaire Average Tool Factor Scores Across Students’ Sociodemographic Characteristics **eTable 2. **Graduation Questionnaire Average Tool Factor Scores Across Students’ Sociodemographic Characteristic

Supplement 2.
Data Sharing Statement
