# Supplementary material for: Development of a Tool to Measure Student Perceptions of Equity and Inclusion in Medical Schools
Source: JAMA Netw Open. 2024 Feb 21;7(2):e240001. doi: 10.1001/jamanetworkopen.2024.0001 (PMC10882418; doi:10.1001/jamanetworkopen.2024.0001)
Supplement: Supplement 1. — eTable 1. Year 2 Questionnaire Average Tool Factor Scores Across Students’ Sociodemographic Characteristics eTable 2. Graduation Questionnaire Average Tool Factor Scores Across Students’ Sociodemographic Characteristic [file jamanetwopen-e240001-s001.pdf]

Supplemental Online Content

Boatright D, Nguyen M, Hill K, et al. A tool to measure student perceptions of equity and inclusion in medical schools. *JAMA Netw Open*. 2024;7(2):e240001. doi:10.1001/jamanetworkopen.2024.0001

eTable 1. Year 2 Questionnaire Average Tool Factor Scores Across Students' Sociodemographic Characteristics
eTable 2. Graduation Questionnaire Average Tool Factor Scores Across Students' Sociodemographic Characteristics

This supplemental material has been provided by the authors to give readers additional information about their work.

© 2024 Boatright D et al. *JAMA Network Open.*

eTable 1. Year 2 Questionnaire Average Tool Factor Scores Across Students' Sociodemographic Characteristics

Mean (SD)	Faculty Role Modeling	Sexual Discrimination & Exploitation	Discrimination, Visible Characteristics	Student Empowerment	Student Fellowship	Cultural Competence	Faculty Interaction	Collaborative Environment
Sex								
Male	88.66 (12.31)	99.64 (4.32)	98.41 (5.96)	62.15 (20.98)	75.00 (16.52)	73.54 (17.60)	70.54 (17.68)	81.22 (17.90)
Female	87.18 (12.28)	99.87 (2.50)	97.31 (6.32)	60.39 (20.49)	75.66 (16.34)	71.64 (19.25)	68.59 (17.82)	79.45 (17.68)
Race and ethnicity								
White	88.35 (11.78)	99.81 (2.94)	98.14 (5.36)	61.65 (20.67)	76.74 (15.73)	74.21 (17.36)	70.65 (17.45)	80.26 (17.47)
Asian	87.24 (13.13)	99.69 (4.29)	97.68 (6.95)	60.52 (20.40)	73.36 (16.90)	71.04 (18.23)	66.88 (17.84)	80.72 (18.09)
Black	84.77 (13.51)	99.65 (4.30)	96.14 (8.56)	60.33 (20.80)	71.62 (17.33)	61.96 (23.15)	67.46 (18.39)	78.95 (18.30)
Hispanic	88.39 (12.69)	99.78 (3.07)	97.38 (6.52)	61.61 (21.82)	71.83 (18.11)	70.76 (20.53)	69.04 (18.86)	80.65 (19.00)
AIAN/HNPI	88.68 (13.38)	99.83 (1.95)	96.41 (7.66)	63.54 (24.20)	72.13 (17.81)	71.15 (21.49)	71.70 (20.11)	81.22 (19.69)
Multiracial	87.87 (12.17)	99.78 (3.32)	97.63 (6.49)	60.65 (20.88)	75.07 (16.90)	72.36 (19.39)	69.78 (17.75)	80.09 (17.88)
Other	87.67 (14.06)	99.21 (6.86)	96.56 (9.24)	58.69 (22.74)	72.33 (18.77)	71.06 (20.98)	67.74 (19.56)	79.97 (20.29)
LGB								
No	88.14 (12.17)	99.79 (3.35)	97.92 (6.04)	61.60 (20.68)	75.46 (16.39)	72.92 (18.15)	69.64 (17.72)	80.60 (17.66)
Yes	84.89 (13.54)	99.39 (4.70)	96.77 (7.50)	56.88 (21.01)	74.00 (16.81)	68.08 (21.74)	68.08 (18.44)	76.65 (18.97)
Low-income								
No	88.00 (12.11)	99.78 (3.39)	98.01 (5.84)	61.51 (20.50)	76.21 (16.05)	73.03 (17.88)	69.71 (17.51)	80.52 (17.44)
Yes	87.59 (12.77)	99.72 (3.69)	97.41 (6.87)	60.55 (21.27)	73.35 (17.09)	71.39 (19.84)	69.05 (18.38)	79.74 (18.60)

**All comparisons were significant with $p<0.001$, Sexual Discrimination and Exploitation was significant at $p=0.04$ for low-income status.*

© 2024 Boatright D et al. *JAMA Network Open.*

eTable 2. Graduation Questionnaire Average Tool Factor Scores Across Students' Sociodemographic Characteristics

Mean (SD)	Faculty Role Modeling	Sexual Discrimination & Exploitation	Discrimination, Visible Characteristics	Student Empowerment	Faculty Support
Sex					
Male	80.98 (13.69)	99.41 (4.07)	96.73 (8.12)	65.16 (21.77)	74.10 (18.78)
Female	79.29 (13.02)	99.61 (2.61)	94.26 (9.95)	63.49 (20.92)	72.24 (18.55)
Race and ethnicity					
White	80.43 (12.81)	99.55 (3.11)	96.27 (7.62)	64.24 (21.18)	74.15 (18.09)
Asian	79.98 (14.38)	99.49 (4.18)	94.80 (10.56)	64.49 (21.51)	71.10 (19.53)
Black	77.12 (14.44)	99.52 (3.40)	91.59 (13.69)	64.00 (21.57)	69.96 (19.57)
Hispanic	80.07 (13.95)	99.37 (3.79)	93.67 (11.28)	65.35 (22.04)	72.47 (19.61)
AIAN/HNPI	79.09 (15.09)	99.12 (6.10)	92.31 (14.36)	63.44 (23.65)	71.50 (20.19)
Multiracial	80.06 (13.20)	99.43 (3.00)	95.01 (9.50)	64.41 (21.45)	73.66 (18.61)
Other	79.92 (15.81)	99.27 (3.70)	92.79 (13.12)	63.16 (23.26)	71.90 (21.41)
LGB					
No	80.36 (13.29)	99.67 (2.95)	95.64 (8.95)	64.64 (21.30)	73.33 (18.63)
Yes	76.89 (14.18)	97.38 (6.97)	93.26 (11.57)	59.74 (21.76)	70.81 (19.33)
Low-income					
No	80.31 (13.11)	99.58 (3.20)	95.90 (8.49)	64.55 (21.15)	73.47 (18.38)
Yes	79.60 (14.15)	99.33 (3.96)	94.25 (10.84)	63.64 (21.96)	72.25 (19.55)

**All comparisons were significant (p<0.01) except for Student Empowerment by race and ethnicity (p=0.08).*

© 2024 Boatright D et al. *JAMA Network Open.*
