# Supplementary material for: Development of a Tool to Measure Student Perceptions of Equity and Inclusion in Medical Schools
Source: JAMA Netw Open. 2024 Feb 21;7(2):e240001. doi: 10.1001/jamanetworkopen.2024.0001 (PMC10882418; doi:10.1001/jamanetworkopen.2024.0001)
Supplement: Supplement 2. — Data Sharing Statement [file jamanetwopen-e240001-s002.pdf]

Data Sharing Statement

Boatright. The Promoting Diversity, Group Inclusion, and Equity (PRODIGIE) Tool for Medical Education. *JAMA Netw Open*. Published February 21, 2024. doi:10.1001/jamanetworkopen.2024.0001

Data
Data available: No
